# Nucleus‐Targeted Organoiridium–Albumin Conjugate for Photodynamic Cancer Therapy

**DOI:** 10.1002/anie.201813002

**Published:** 2019-01-21

**Authors:** Pingyu Zhang, Huaiyi Huang, Samya Banerjee, Guy J. Clarkson, Chen Ge, Cinzia Imberti, Peter J. Sadler

**Affiliations:** ^1^ College of Chemistry and Environmental Engineering Shenzhen University Shenzhen 518060 China; ^2^ School of Pharmaceutical Science (Shenzhen) Sun Yat-sen University Guangzhou 510275 China; ^3^ Department of Chemistry University of Warwick Coventry CV4 7AL UK

**Keywords:** albumin, organoiridium, photodynamic therapy, photosensitizers

## Abstract

An organoiridium–albumin bioconjugate (**Ir1‐HSA**) was synthesized by reaction of a pendant maleimide ligand with human serum albumin. The phosphorescence of **Ir1‐HSA** was enhanced significantly compared to parent complex **Ir1**. The long phosphorescence lifetime and high ^1^O_2_ quantum yield of **Ir1‐HSA** are highly favorable properties for photodynamic therapy. **Ir1‐HSA** mainly accumulated in the nucleus of living cancer cells and showed remarkable photocytotoxicity against a range of cancer cell lines and tumor spheroids (light IC_50_; 0.8–5 μm, photo‐cytotoxicity index PI=40–60), while remaining non‐toxic to normal cells and normal cell spheroids, even after photo‐irradiation. This nucleus‐targeting organoiridium‐albumin is a strong candidate photosensitizer for anticancer photodynamic therapy.

Photodynamic therapy (PDT) is a non‐invasive cancer therapy,^1^ which uses photosensitizers and light to convert cellular triplet oxygen (^3^O_2_) into highly reactive and cell‐damaging singlet oxygen (^1^O_2_).^2^ Examples of clinical photosensitizers include hematoporphyrin derivatives (Photofrin) and aminolevulinic acid (ALA, a porphyrin precursor).^3^ In recent years, metal complexes with high luminescence have emerged as promising photo‐theranostic candidates owing to their superior photochemical and photophysical properties.^4^ An octahedral tris‐*N*,*N*‐chelated Ru^II^ complex (TLD1433) recently entered clinical trials for bladder PDT,4a and WST 11 (TOOKAD Soluble), a square‐planar Pd^II^ bacteriochlorophyll derivative, has been approved for vascular‐targeted PDT.4b

Human serum albumin (HSA) conjugates can be used to delivery anticancer drugs. HSA is abundant in blood serum (ca. 0.6 mm), rich in histidine, and contains a free thiol residue at cysteine‐34.^5^ Moreover, HSA functions as a physiological antioxidant,^6^ and binds a wide range of biologically and clinically important molecules.^7^ The effectiveness of HSA‐coupled anticancer drugs has been established clinically for doxorubicin (INNO206; aldoxorubicin),^8^ and HSA‐based nanoparticle‐encapsulated paclitaxel (Abraxane).^9^ Recently, HSA‐functionalized metal complexes (for example, Pt, Ru, Os) have been developed for cancer therapy, illustrating that HSA plays a key role in augmenting anticancer activity.^10^

Herein we have conjugated a maleimide‐functionalized octahedral organo‐iridium(III) complex (**Ir1**, Figure 1 a) to HSA, giving rise to a large enhancement in the phosphorescence of **Ir1‐HSA** compared to **Ir1. Ir1‐HSA** is notably nontoxic in the dark, but exhibits potent photo‐cytotoxicity with significant selectivity for cancer cells and cancer cell spheroids over normal cells. **Ir1‐HSA** appears to be the first example of an HSA‐functionalized iridium conjugate which allows targeting of cell nuclei, as well as being an efficient photosensitizer for PDT.

**Figure 1:**
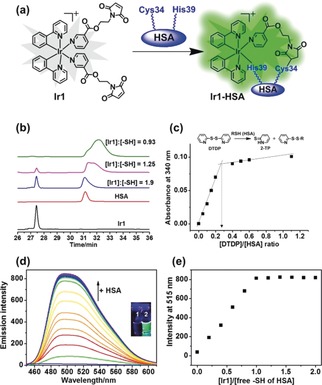
a) The conjugation of **Ir1** to HSA. b) Reaction of **Ir1** (30 μm) with various concentrations of HSA (0, 60, 90, and 120 μm, concentrations of Cys34 free thiol group were 0, 16.2, 24.3, 32.4 μm, respectively) studied by RP‐HPLC (UV detection at 280 nm). c) Variation of absorbance at 340 nm at various HSA:2,2′‐DTDP ratios ([HSA]=80 μm; [DTDP]=0–110 μm) and determination of the thiol content of HSA. d) Emission spectra of **Ir1** (4 μm) in the presence of increasing concentrations of HSA (0–30 μm, [Cys34 free thiol] 0–8.1 μm), in PBS (pH 7.4), reaction time: 20 min at each concentration, *λ*_ex_=405 nm; Inset: photos of **Ir1** (1) and **Ir1‐HSA** (2) under UVA irradiation. e) Dependence of the phosphorescence intensity of **Ir1** at 515 nm on the concentration of free thiol of HSA, as ratio [**Ir1**]/[free thiol].

The octahedral organo‐iridium(III) complex **Ir1**, containing two chelated phenylpyridine ligands and two monodentate pyridines functionalized with a maleimide substituent, was synthesized and fully characterized as described in the Supporting Information. It was highly stable in phosphate‐buffered saline (PBS) solution for 12 h in the dark and photostable after 1 h irradiation with blue light (465 nm; Supporting Information, Figure S1).

To investigate the reactivity of the C=C bond of the pendant maleimides of **Ir1**, the complex was reacted with cysteine (Cys) in a molar ratio of 2[Cys] to 1[**Ir1**] in [D_6_]DMSO/D_2_O (2/1 v/v) at 298 K for 30 min. ^1^H NMR peaks for the vinyl protons of the maleimide groups at 6.62 ppm disappeared upon the addition of Cys, and new peaks appeared between 2.9 ppm and 3.9 ppm assignable to conjugated Cys (Supporting Information, Figure S2). The intensities of the peaks indicated that Cys reacted with each of the two pendant maleimides of **Ir1**. The conjugate was further characterised by ESI‐MS (Supporting Information, Figures S3, S4).

To investigate whether the free Cys34 thiol of HSA can similarly react with the C=C in maleimide, **Ir1** (30 μm) was incubated with various amounts of HSA (0–120 μm) for 1 h and the products were separated by RP‐HPLC. The peak for **Ir1** gradually disappeared with increasing amounts of HSA, with complete reaction observed at 120 μm HSA (Figure 1 b). This molar ratio of [HSA]:[**Ir1**] of 4:1, is consistent with the free thiol content of the HSA used, determined via reaction with 2,2′‐dithiodipyridine (2,2′‐DTDP) using a slightly modified previously reported approach.^11^ This gave a thiol content of 0.27±0.1 mol SH per mol HSA (Figure 1 c). Hence, the concentration of free −SH groups from 120 μm HSA was 32.4±1.2 μm, which reacted with 30 μm **Ir1**, suggesting formation of a 1:1 **Ir1**:HSA adduct. As Cys34 is in a crevice, it is likely that the second maleimide group is not accessible to a second HSA. RP‐HPLC studies on the time‐dependent binding showed that the reaction was relatively rapid with a half‐life of ca. 20 min (Supporting Information, Figure S5).

**Ir1** (4 μm) itself was only weakly emissive in aqueous solution (*λ*_ex_=405 nm), whereas **Ir1‐HSA** showed strong phosphorescence under the same conditions (inset graph Figure 1 d). The phosphorescence of **Ir1** gradually increased with increasing concentrations of HSA (Figure 1 d), plateauing at a mol ratio of ca. 1.0 **Ir1**:HSA(free‐SH), with a phosphorescence enhancement of ca. 53‐fold (Figure 1 e).

To remove free thiol groups from HSA (by oxidation to a disulfide), a 10‐fold molar excess of cystine was added to a 100 μm HSA solution for 24 h at 277 K. Then this HSA‐Cys34‐S‐S‐Cys product was reacted with **Ir1** for 30 min. The observed phosphorescence was much weaker compared to the conjugate formed by reaction of HSA‐Cys34 and **Ir1** (Figure 2 a), clearly suggesting that the free thiol of Cys34 is the binding site for **Ir1**.

**Figure 2:**
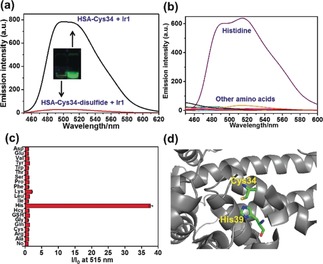
a) Emission intensity of HSA (100 μm) after reaction with 10 mol equiv cystine (giving disulfide formation at Cys34) in PBS (pH 7.4) for 24 h at 277 K, followed by treatment with **Ir1** for 30 min; Inset: Images of the reaction mixture compared to **Ir1‐HSA** under UVA irradiation, showing the decrease in phosphorescence. b) Emission intensity of **Ir1** (4 μm) in the presence of various amino acids (100 μm) in PBS solution. c) Emission intensity at 515 nm of **Ir1** in the presence (*I*) vs. the absence (*I*_0_) of amino acids according to (b). d) Structure of HSA (PDB:5IJF); Cys34 and His39 are labeled.

HSA is a large protein (66.5 kDa) with a single‐chain of 585 amino acid residues.^5^, ^6^, ^7^ To provide an indication of which residues of HSA might be involved in the luminescence enhancement of **Ir1**, the interaction of **Ir1** with various amino acids was studied by luminescence analysis (Figure 2 b,c). Interaction of **Ir1** with histidine resulted in an emission enhancement of about 37‐fold. In contrast, no significant luminescence enhancement was observed with the other amino acids, including Cys (Figure 2 b,c). Although **Ir1** binds to Cys34, other factors appear to be responsible for the enhancement of phosphorescence of **Ir1‐HSA**. These include interactions with His residues, a strong candidate being nearby His39 (Figure 2 d).

Ir^III^ complexes with weakly bound ligands are known to bind strongly to amino acids/proteins through ligand substitution reactions, especially to histidine/histidine rich proteins, and their use in protein staining has been reported.^12^ Here it is evident that histidine can switch on the phosphorescence of **Ir1**. We recorded the ESI‐MS of a mixture of **Ir1** and His; two peaks at *m*/*z* 656.2 and 903.2 assignable to His‐bound Ir^III^ species were detected (Supporting Information, Figure S6). These results suggest that one sterically‐hindered monodentate maleimide ligand is released after binding of **Ir1** to Cys34, being displaced by His39.

As **Ir1‐HSA** emitted strong phosphorescence, we evaluated **Ir1‐HSA** as a potential photosensitizer for PDT. Firstly, **Ir1** (0.4 mm) was dissolved in 20 mL MeOH:H_2_O (1:2 v/v), and HSA (0.4 mm) was added. The reaction mixture was stirred for 1 h, followed by **Ir1‐HSA** purification by dialysis (10 kDa cut‐off filter) to remove unbound **Ir1**. The **Ir1‐HSA** conjugate showed a new IR band at 1043 cm^−1^, characteristic of a new C−S stretch (Supporting Information, Figure S7).

Next the localization of **Ir1‐HSA** in living A549 lung cancer cells was investigated using confocal microscopy. The cells were incubated with **Ir1‐HSA** ([Ir]=5 μm) for various times. Real‐time imaging showed that **Ir1‐HSA** mainly accumulated in the cytoplasm within the first 30 min and then migrated to the nucleus upon further incubation (60 min–120 min; Figure 3). In contrast, the green luminescence of **Ir1** alone was observed throughout the cells, both in cytoplasm and nucleus (Supporting Information, Figure S8). This subcellular localization of **Ir1** and **Ir1‐HSA** in A549 cells was also confirmed by ICP‐MS (Supporting Information, Figure S9), demonstrating that iridium localized both in the cytoplasm and nucleus of cells treated with **Ir1**, whereas it mainly accumulated in the nucleus of **Ir1‐HSA** treated cells. We further confirmed that the luminescence of **Ir1‐HSA** was perfectly co‐localized with the blue fluorescence of Hoechst 33 258 (a nucleus dye), with a co‐localization efficiency of 0.82 in live A549 cells (Supporting Information, Figure S10).

**Figure 3:**
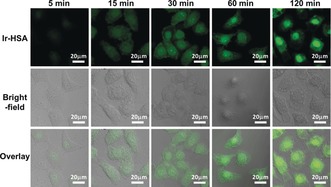
Confocal microscopy images of living A549 cells incubated with **Ir1‐HSA** ([Ir]=5 μm) in real time (5, 15, 40, 60, 120 min); *λ*_ex_=405 nm, *λ*_em_=550±20 nm.

To address the question as to whether HSA co‐migrated with Ir, we used an immunofluorescence approach to determine the intracellular localization of HSA. As expected, HSA was not detected in **Ir1**‐treated A549 cells (Figure 4). However, **Ir1‐HSA** exposure led to the accumulation of HSA in the cytoplasm and the nuclear membrane. When the cells were treated with HSA, the red luminescence was observed as well. This suggests that, although HSA facilitates delivery of the iridium complex to the nucleus, it does not itself penetrate past the nuclear membrane, and is probably released from **Ir1‐HSA** before the Ir^III^ complex migrates into the nucleus.

**Figure 4:**
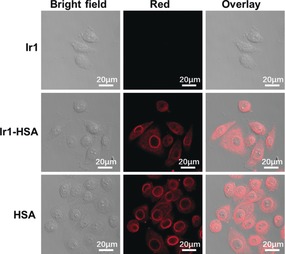
Immunofluorescence staining of HSA in cells exposed to **Ir1**, HSA, **Ir1‐HSA** ([Ir]=5 μm, 2 h), respectively. *λ*_ex_=563 nm; *λ*_em_=580–630 nm.

The phosphorescence quantum yield of **Ir1** was very low (0.001) and its phosphorescence lifetime only 182.7 ns in methanol/PBS (1:1, v/v; Figure 5 a; Supporting Information, Table S1) at 298 K. Compared to **Ir1**, the quantum yield for **Ir1‐HSA** increased 36× (to 0.036) and its emission lifetime to 871.8 ns. The long phosphorescence lifetime of **Ir1‐HSA** makes it ideal for ^1^O_2_ generation. We used electron paramagnetic resonance (EPR) spectroscopy with 2,2,6,6‐tetramethylpiperidine (TEMP) as a spin trap to detect ^1^O_2_ generation by **Ir1** and **Ir1‐HSA** under 465 nm irradiation. As illustrated in Figure 5 b, a characteristic 1:1:1 triplet assignable to 2,2,6,6‐tetramethylpiperidine‐1‐oxyl was observed upon irradiation (20 min). Notably the intensity of the EPR signal generated by **Ir1‐HSA** was significantly stronger than for **Ir1**. The ^1^O_2_ quantum yield^13^ (Φ(^1^O_2_)) of **Ir1‐HSA** was 0.83, much higher than that for **Ir1** (0.06) upon 465 nm light irradiation (Supporting Information, Table S1).

**Figure 5:**
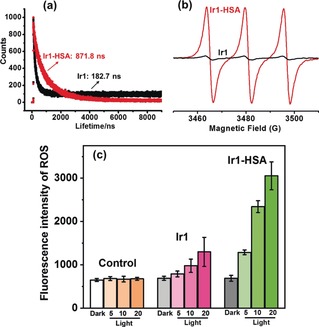
a) Phosphorescence lifetimes of **Ir1** and **Ir1‐HSA** in methanol/PBS (1:1 v/v) at 298 K; b) EPR spectra of **Ir1** and **Ir1‐HSA** with TEMP after 20 min light irradiation (465 nm, 5.76 J cm^−2^). c) Fluorescence intensity of ROS in A549 cells treated with **Ir1** or **Ir1‐HSA** ([Ir]=5 μm) and ROS probe in the dark or upon light irradiation (5, 10, 20 min). The excitation wavelength of the ROS probe was 520 nm and the fluorescence was measured at 590–625 nm.

The long excited‐state lifetime and very high ^1^O_2_ generation quantum yield make **Ir1‐HSA** a potential PDT agent. To explore this, the dark‐ and photo‐antiproliferative activity of **Ir1** and **Ir1‐HSA** was determined against human cancer cells (lung: A549; hepatoma: Hep‐G2; cisplatin resistant lung: A549R) and normal human cells (lung: MRC‐5; liver: LO2). Cells in the light plate were incubated with **Ir1** or **Ir1‐HSA** for 2 h in the dark, washed with PBS, followed by 20 min irradiation using blue LEDs (465 nm, 5.76 J cm^−2^), while the dark plate was kept in the dark. Then all cells were allowed to recover over 46 h. No cell death was observed for untreated cells exposed to light (Supporting Information, Figure S11). **Ir1** was relatively nontoxic toward A549 cells both in the dark (89.6 μm) and light (53.3 μm) (Table 1). In contrast, **Ir1‐HSA** was non‐toxic towards A549 cells in the dark (62.3 μm), but became highly cytotoxic upon irradiation (1.1 μm) with a high photocytotoxicity index (PI, PI=dark IC_50_/light IC_50_) of 56.6 (Supporting Information, Figure S11). Similar photodynamic efficiency for **Ir1‐HSA** was also observed for Hep‐G2 and A549R cells. Notably, under the same experimental conditions, both **Ir1** and **Ir1‐HSA** were non‐toxic toward normal cells (MRC‐5 and LO2) (Table 1).

**Table 1:** IC_50_ (μm) values for **Ir1** and **Ir1‐HSA** against 2D and 3D (spheroids) cancer and normal cell lines.

Cell lines^[a]^	**Ir1**		**Ir1‐HSA**	
	Dark	Light	Dark	Light
A549	89.6^±3.7^	53.3^±4.5^	62.3^±2.6^	1.1^±0.3^
Hep‐G2	83.5^±3.7^	54.8^±2.6^	85.6^±3.2^	2.2^±0.3^
A549R	75.6^±4.1^	56.2^±1.5^	84.9^±5.9^	2.3^±0.2^
MRC‐5	90.6^±1.7^	76.9^±1.6^	96.4^±6.1^	78.7^±2.3^
LO2	89.3^±2.3^	76.5^±0.9^	88.6^±3.0^	66.4^±4.5^
A549 spheroid	>100	>100	65.6^±5.9^	4.8^±0.2^
MRC‐5 spheroid	>100	>100	>100	>100

[a] Cells were incubated with the compounds for 2 h in the dark, washed, fresh medium added, followed by incubation in the dark or irradiation at 465 nm (20 min, 5.76 J cm^−2^), and a further 46 h incubation. IC_50_ values for **Ir1** and **Ir1‐HSA** are based on Ir concentration. Under the same experimental conditions, 5‐aminolevulinic acid, a clinical PDT agent and cisplatin gave IC_50_>100 μm both in the dark and upon light irradiation. The IC_50_ values (concentrations which caused 50 % of cell death) were determined as duplicates of triplicates in three independent sets of experiments. For each data point, the average and standard deviation are reported. For 3D toxicity assays, 8 spheroids were selected for each condition studied.

We further investigated the photocytoxicity in 3D multicellular spheroids (MCSs) with semidiameters of about 400 μm. The cytotoxicities of the complexes toward MCSs were determined by measurement of ATP concentrations using the CellTiter‐Glo 3D Cell Viability Assay (Promega). As shown in Table 1, both **Ir1** and **Ir1‐HSA** were non‐toxic towards A549 cancer spheroids and normal cell spheroids in the dark (IC_50_>50 μm). However, **Ir1‐HSA** showed strong phototoxic effects on A549 cancer spheroids upon light irradiation, with an IC_50_ value of 4.8 μm. We also studied the effect of **Ir1‐HSA** on the kinetics of 3D MCSs regrowth. After treatment with **Ir1‐HSA** ([Ir]=5 μm) in the dark, the diameters of 3D MCSs increased slightly after 48 h. However, the MCSs treated with **Ir1‐HSA** followed by 20 min light irradiation decreased in size over time (Supporting Information, Figure S12).

A reactive oxygen species (ROS) detection assay kit was used to determine whether **Ir1‐HSA** produced ROS within the cells upon irradiation. Cells treated with the red ROS probe and **Ir1** or **Ir1‐HSA** in the dark showed no evident fluorescence and there was only a very weak fluorescence in the cells treated with **Ir1**. In contrast, a strong red fluorescence was shown within the cells pre‐treated with **Ir1‐HSA** following light irradiation (Figure 5 c), suggesting that **Ir1‐HSA** generated ROS efficiently in cancer cells upon light irradiation.

In summary, we have reported the first example of an organo‐iridium complex‐HSA bioconjugate as a nucleus‐targeted vehicle for anticancer photodynamic therapy. The phosphorescence of **Ir1** was greatly enhanced by conjugation to HSA. Interestingly, **Ir1‐HSA** accumulated mostly in the nucleus of living cancer cells. In contrast to other well‐studied cyclometalated iridium complexes, which mainly located in the cytoplasm, **Ir1‐HSA** appears to be the first reported nucleus‐targeting photosensitizer. There are only a few reports of the transport of albumin to the nucleus: in response to oxidative stress,^14^ and via permeabilization of cells with digitonin.^15^ In our work, it appears that albumin plays an important role in the transport and delivery of **Ir1** to the cell nucleus.

Importantly, **Ir1‐HSA** exhibited a long phosphorescence lifetime and remarkably high ^1^O_2_ generation quantum yield along with high photostability, which are essential for an efficient photosensitizer. **Ir1‐HSA** exhibited excellent photocytotoxicity against a range of cancer cell lines and multicellular spheroids with a high photo‐cytotoxicity index while remaining dormant in normal cells/spheroids, even after photo‐irradiation. All these properties confirm that **Ir1‐HSA** could be an efficient photosensitizer with novel nucleus‐targeting properties for potential clinical PDT applications.

## Conflict of interest

The authors declare no conflict of interest.

## Supporting information

As a service to our authors and readers, this journal provides supporting information supplied by the authors. Such materials are peer reviewed and may be re‐organized for online delivery, but are not copy‐edited or typeset. Technical support issues arising from supporting information (other than missing files) should be addressed to the authors.

Supplementary
