# Supplementary material for: Nucleus‐Targeted Organoiridium–Albumin Conjugate for Photodynamic Cancer Therapy
Source: Angew Chem Int Ed Engl. 2019 Jan 21;58(8):2350–4. doi: 10.1002/anie.201813002 (PMC6468315; doi:10.1002/anie.201813002)
Supplement: Supplementary file 1 — Supplementary [file ANIE-58-2350-s001.pdf]

Angewandte Chemie
Eine Zeitschrift der Gesellschaft Deutscher Chemiker

Supporting Information

Nucleus-Targeted Organoiridium–Albumin Conjugate for Photodynamic Cancer Therapy

Pingyu Zhang, Huaiyi Huang, Samya Banerjee, Guy J. Clarkson, Chen Ge, Cinzia Imberti, and Peter J. Sadler**

anie_201813002_sm_miscellaneous_information.pdf

Supporting Information

Table of Contents

Experimental section

Materials

Instruments

Synthesis of the maleimide ligand.

Synthesis of Ir(III) complex.

Determination of free thiol content of HSA.

Synthesis of **Ir1-HSA** conjugate.

Electron paramagnetic resonance experiment.

Cellular localization assay.

Immunofluorescence staining of HSA in A549 cells.

Photocytotoxicity towards 2D cells and 3D spheroids.

Detection of intracellular $^1O_2$.

Table and Figures

Table S1. Photophysical properties of **Ir1** and **Ir1-HSA**.

Fig. S1. Stability of **Ir1** in the dark and under light irradiation.

Fig. S2. ^{1}H NMR spectra of **Ir1** upon addition of Cys.

Fig. S3. ESI-MS spectra of **Ir1** and the products **from** reaction of **Ir1** with Cys.

Fig. S4. HR-MS analysis of the products of **Ir1** reaction with Cys.

Fig. S5. Time dependent HPLC traces for the reaction mixture containing **Ir1** and HSA

Fig. S6. ESI-MS spectrum of the products from reaction of **Ir1** with histidine.

Fig. S7. IR spectra of HSA, **Ir1** and **Ir1-HSA**.

Fig. S8. Confocal microscopy images of A549 cells treated with **Ir1** in real time.

Fig. S9. Iridium concentrations in membrane, nucleus and cytoplasm of A549 human lung cancer cells after exposure to **Ir1** and **Ir1-HSA**.

Fig. S10. Confocal images of A549 cells co-stained with **Ir1-HSA** and Hoechst 33258.

Fig. S11. Brightfield images of A549 cells in the dark and after irradiation.

Fig. S12. Growth curves for A549 3D MCSs treated with **Ir1-HSA**.

References

Experimental Section

Materials

HSA, CT-DNA, other proteins and amino acids were purchased from Sigma-Aldrich. The HSA was 96% purified and fatty acid free. The concentration of HSA was determined by absorption spectroscopy ([HSA] = A_{280}/36850 M, 1 cm cuvette). The guanidine hydrochloride was from Alfa Aesar. The cell lines including human lung carcinoma (A549), hepatoma cell line (Hep-G2), cisplatin resistant lung cancer cell line (A549R), human normal lung fibroblast cell line (MRC-5) and human normal liver cell line (LO2) were purchased from Sigma-Aldrich. They were cultured in Roswell Park Memorial Institute medium (RPMI-1640, without phenol red in photo-experiments) with glutamine and penicillin/streptomycin.

Instruments

NMR spectra were recorded on a Bruker AV-400/300 spectrometer. Elemental analysis was performed by Exeter Analytical using a CHN/O/S Elemental Analyser (CE440). Positive ion ESI-MS spectra were obtained using an Agilent 6130B single quad coupled to an automated sample delivery system (isocratic Agilent 1100 HPLC without column). UV-visible absorption spectra were recorded in 1-cm cuvettes on a Varian Cary 300 UV-vis spectrophotometer. The fluorescence spectra were recorded on a JASCO FP-6500 Fluorimeter (band width (ex): 5 nm; band width (em): 5 nm; λ_{ex} = 405 nm; λ_{em} = 450-650 nm). The EPR measurements were carried out at ambient temperature on a Bruker EMX spectrometer. The IR spectra were recorded on a PerkinElmer Fourier Transform Infrared Spectrometer. The confocal images were visualized using Zeiss 710 or 880 confocal microscopy (63× oil-immersion objective).

Synthesis of the Maleimide Ligand (E)

Overall synthetic scheme:

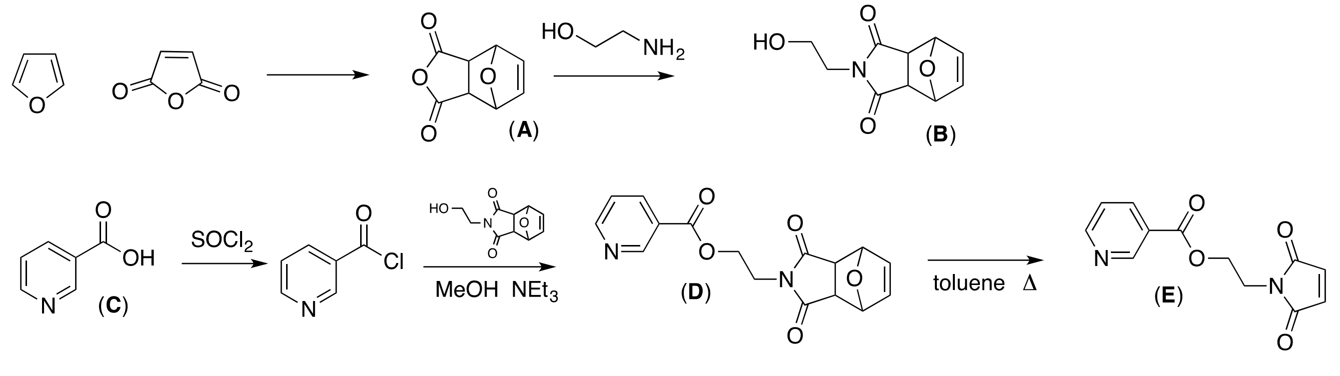

Furan Protected Maleimide Alcohol (B): Synthesized according to a modified literature procedure.[1] Anhydride (**A**) (5 g, 28 mmol) was dissolved in ethanol (50 mL). The solution was cooled to 0 °C and a solution of ethanolamine (1.8 mL, 30 mmol) and NEt_3 (4.2 mL, 30 mmol) in ethanol (10 mL) was added dropwise to the reaction mixture and left to stir for 30 min. It was then heated overnight at 70 °C. Solvent was partially removed *in vacuo* until the product began to precipitate. It was then cooled to 0 °C for 10 minutes and filtered and washed with ice cold ethanol to provide a light pink solid (5.43 g, 26 mmol, 84%). ^1H NMR (300 MHz, CD_3CN): δ_H 6.51 (s, 2H), 5.12 (s, 2H), 3.51 (t, *J* = 6.0 Hz, 2H), 3.54 (t, *J* = 6.0 Hz, 2H), 2.90 (s, 2H), 2.24 (s, 1H). ^{13}C NMR (75 MHz, CD_3CN): δ_C 171.0 (*C*=O), 136.1 (*C*H=*C*H), 80.5 (bridge *C*HO), 58.0 (CH_2*C*H_2O), 47.1 (*C*HCO), 40.5 (CH_2*C*H_2N). IR ν cm^{-1}: 3472 (OH), 1682 (imide C=O). MS (ESI^+): *m/z* 231.9 $[M+Na]^+$.

Furan Protected Maleimide Nicotinate (D): To a mixture of nicotinic acid **C** (2.0 g, 16 mmol) in thionyl chloride (20.0 mL, excess) 3 drops of DMF were added carefully. The mixture was stirred at room temperature for overnight and then the thionyl chloride was removed under low pressure (at 40°C) to give a white powder which was used without further purification. To a mixture of the nicotinoyl chloride in CH_2Cl_2 (20 mL), a solution of alcohol **B** (3.74 g, 1.1 mol equiv, 17.9 mmol) and NEt_3 (5 mL, 35.9 mmol) in CH_2Cl_2 (15 mL) was added dropwise over 10 min. The mixture was stirred for 48 h. To the reaction mixture, a saturated solution of aq sodium carbonate (50 mL) was added and allowed to stir for another 20 min. The mixture was separated and the organic layer was washed with sodium hydrogen carbonate (2 x 50 mL), dried over $MgSO_4$, filtered and the solvent removed under reduced pressure to provide a white solid which was recrystallised from isopropanol (2.97 g, 9.46 mmol, 58%). m.p. 83-84°C; ^1H NMR (400 MHz, $CDCl_3$): δ_H 9.14 (s, 1H), 8.87 (d, 1H, *J* = 7.0 Hz), 8.23 (d, 1H, *J* = 7.0 Hz), 7.35 (t, 1H, *J* = 7.0 Hz), 6.50 (s, 2H), 5.22 (s, 2H), 4.48 (t, 2H, *J* = 5.0 Hz), 3.90 (t, 2H, *J* = 5.0 Hz), 2.86 (s, 2H); ^{13}C NMR (100 MHz, $CDCl_3$): δ_C 176.0 (imide *C*=O), 165.0 (*C*OO), 153.6 (Pyr*C*H), 151.1 (Pyr*C*H), 137.1 (Pyr*C*H), 136.5 (*C*H=*C*H), 125.7 (Pyr*C*), 123.3 (Pyr*C*H), 80.9 (bridge *C*HO), 61.6 (CH_2*C*H_2O), 47.5 (2 endo *C*HCO), 37.7 (CH_2*C*H_2N). IR ν cm^{-1}: 3021.9 (C-H stretching), 1688.3 (C=O stretching). HR-MS (ESI^+): *m/z* found, 315.0978 calculated for $C_{16}H_{15}N_4O_5$ 315.0981 ($[M+H]^+$).

Maleimide Nicotinate (E): Furan-protected maleimide nicotinate **D** (0.5 g, 1.6 mmol) was refluxed in toluene and the reaction monitored by TLC (EtOAc). On consumption of starting material (up to 24 h), the reaction was allowed to cool and the solvent was removed *in vacuo* to give a beige solid that was recrystallized from EtOAc/petrol (315 mg, 1.28 mmol, 80%). ^1H

NMR (400 MHz, $CDCl_3$): δ_H 9.15 (s, 1H), 8.76 (d, *J* = 5.0 Hz, 1H), 8.25 (d, *J* = 8.0 Hz, 1H), 7.38 (dd, *J* = 5.0, 8.0 Hz, 1H), 6.74 (s, 2H), 4.49 (t, *J* = 5.0 Hz, 2H), 3.95 (t, *J* = 5.0 Hz, 2H). ^{13}C NMR (100 MHz, $CDCl_3$): δ_C 170.4 (imide *C*=O), 165.1 (*C*OO), 153.6 (Pyr*C*H), 151.1 (Pyr*C*H), 137.2 (Pyr*C*H), 134.3 (*C*H=*C*H), 125.6 (Pyr*C*), 123.4 (Pyr*C*H), 62.6 (CH_2*C*H_2O), 36.8 (CH_2*C*H_2N). MS (ESI^+): *m/z* 247.0 $[M+H]^+$. Anal. Calcd for $C_{12}H_{10}N_2O_4$: C, 58.54; H, 4.09; N, 11.38%. Found: C, 58.56; H, 4.08; N, 11.41%.

Synthesis of Ir(III) Complex (Ir1)

The iridium dimer $[(ppy)_4Ir_2Cl_2]$[2] (0.05 mmol, 1 mol eq) and the maleimide ligand (0.3 mmol, 6 mol equiv) were placed in a round bottom flask and DCM and methanol (1:1, 30 mL) was added. The mixture was stirred for 10 h at room temperature. After the reaction, solvents were reduced to 3 mL, NH_4PF_6 was added, the yellow solid was filtered and washed with water and diethyl ether. The solid was further purified by silica gel column chromatography (eluted with CH_2Cl_2 : methanol = 80 : 20) to give the pure complex. Yield: 83%. ^{1}H NMR (300 MHz, DMSO): δ_H = 9.56 (d, *J* = 5.5 Hz, 2H), 9.03 (d, *J* = 10.4 Hz, 2H), 8.79 (d, *J* = 4.4 Hz, 2H), 8.17 – 8.03 (m, 4H), 7.99 (t, *J* = 7.6 Hz, 2H), 7.73 (dd, *J* = 15.7, 7.5 Hz, 4H), 7.42 (t, *J* = 6.6 Hz, 2H), 7.01 (s, 4H), 6.71 (dt, *J* = 14.9, 7.7 Hz, 4H), 6.27 (d, *J* = 7.6 Hz, 2H), 4.42 (dd, *J* = 15.1 Hz, 10.3, 4H), 3.83 (t, *J* = 4.6 Hz, 4H). Anal. Calcd for $C_{46}H_{36}IrN_6O_8PF_6$: C, 51.21; H, 3.33; N, 7.79%. Found: C, 51.19; H, 3.34; N, 7.75%. ESI-MS: 933.2.

Determination of Free Thiol Content of HSA

The concentration of free thiol in HSA was determined via reaction with 5,5'-dithiobis(2-nitrobenzoic acid) (DTNB) or 2,2'-dithiodipyridine (2,2'-DTDP) using a slightly modified literature approach.[3] Reaction of the thiol results in the stoichiometric formation of 2-thiopyridone (2-TP) after 40 min incubation time. The molar ratio method was used to determine the stoichiometry of the reaction corresponding to the concentration of the thiol groups. The protein concentration was kept constant and the HSA:2,2'-DTDP ratio was varied. Then, the absorbance of the generated 2-TP was determined and plotted against the concentration of the reagent.

Synthesis of Ir1-HSA Conjugate

The iridium complex (0.4 mmol) was dissolved in 20 mL methanol and water (v/v 1:2). Then HSA (0.4 mmol) in water was added. The mixture was stirred for 1 h and purified by dialysis (10 kDa MWCO membrane). The purified **Ir1-HSA** conjugate was collected and freeze dried. The concentration of Ir was determined by Inductively-Coupled-Plasma Optical Emission

Spectroscopy (ICP-OES).

Electron Paramagnetic Resonance (EPR) Experiment

An LED lamp (465 nm) was used as light source. The sample was contained in a flat-cell (WG812) positioned in a TM110 cavity (ER4103 TM). The EPR parameters were: sweep width 8 mT, 1024 points, time constant 10.24 ms and conversion time 20.48 ms, giving a sweep time of ~30 s. Field modulation was applied at 100 kHz and 0.05 mT, and the microwave attenuation was 18 dB (~3.2 mW). The spin trap for $^1O_2$ 2,2,6,6-tetramethyl-piperidine (TEMP, 20 mM) was used to verify the formation of reactive oxygen species (ROSs) generated by the compounds (20 μM) under 465 nm light irradiation for 20 min.

Cellular Localization Assay

The living/fixed A549 cancer cells (the fixed cells were treated with 4% paraformaldehyde) were incubated with **Ir1/Ir1-HSA** ([Ir] = 5 μM) alone for 2 h or co-stained with Hoechst 33258 (500 nM) for 30 min, washed three times with PBS and visualized using Zeiss confocal microscopy (63× oil-immersion objective). For the iridium complex **Ir1**, the excitation wavelength was 405 nm; the emission wavelength was 550 ± 20 nm; For Hoechst 33258, the excitation wavelength was 405 nm; the emission wavelength was 450 ± 20 nm.

The cellular localization was also determined by ICP-MS. The cells were plated at a density of 1×10^6 cells/mL in cultured dishes. **Ir1** or **Ir1-HSA** ([Ir] = 5 μM) was added to the culture medium and incubated for 2 h, and the cells collected. Then the membrane, nucleus and cytoplasm fractions of the cells were extracted by using appropriate extraction kits (Promega). The samples were digested overnight in 60% HNO_3 at ambient temperature for 24 h. Each sample was then diluted with Milli-Q water to give a 3% HNO_3 sample solution. The iridium content was determined by inductively-coupled-plasma mass spectrometry (ICP-MS, Agilent 7500 series).

Immunofluorescence Staining of HSA in A549 Cells

10^6 A549 cells were cultured in 6-well plates and treated with HSA, **Ir1** or **Ir1-HSA** ([Ir] = 5 μM) for 2 h in an incubator. Then the cells were washed with PBS and fixed with 4% paraformaldehyde for 20 min, then permeabilized with 0.3% Triton X-100 for 10 min, blocked with 10% fetal bovine serum in PBS for 1 h, and incubated with rabbit anti-human HSA monoclonal antibody (15 μg/mL) at 37 °C for 1 h. Subsequently, the cells were washed 5x with PBS and incubated with Cy3-labeled goat anti-rabbit IgG (H+L) (λ_{ex} = 563 nm; λ_{em} = 580-630

nm) for 1 h at 37 °C. The images were visualized under a 63× oil-immersion objective using a Zeiss confocal microscope.

Photocytotoxicity towards 2D Cells and 3D Spheroids

Cytotoxicities in the dark and light were determined for **Ir1** and **Ir1-HSA**. Black or white 96-well plates were seeded with ca. 5,000 cells per well, and then incubated for 24 h. Varying concentrations (0-100 μM) of the complexes (0.5-2% (v/v) DMSO) were added to the cells, incubated for 2 h, washed with PBS and fresh RPMI-1640 medium without phenol red was added to the wells. The plates used for photocytotoxicity determination were then irradiated using 465 nm light for 20 min. After irradiation, the cells were incubated for a further 46 h in complex-free medium, washed with PBS, and 50 μL of 50% TCA was added. The plates were left in a fridge at 4 °C for 2 h after which they were washed and dried. The cell viability was then determined by the SRB assay.[4] SRB dye (0.4% wt/v in 1% acetic acid) was added to the cells and left at ambient temperature. The plates were then washed vigorously with 1% acetic acid. Tris-base (pH 10.5, 10 mM) was added and the cells were left for 30 min. The change in absorbance was measured at 510 nm using a Glo-max Multi Microplate Reader (Promega). All the IC_{50} values (concentrations which caused 50% of cell death) were determined as duplicates of triplicates in three independent sets of experiments. The raw data were fitted as a sigmoidal dose response using the Origin software package. The equation used is shown below[5]:

$$y = y_i + \frac{y_i - y_f}{1 + 10^{(\log IC_{50} - x)\mathrm{Hillslope}}}$$

where y_i and y_f are the initial and final signal intensities. The standard deviations were calculated for the three independent experiments using the STDEV function in Excel software.

Three-dimensional (3D) multicellular spheroids (MCSs) were cultured using the liquid overlay method. Approximately 5,000 diluted cells were transferred to cell-repellent surface U-shaped 96-well plates (Greiner Bio-One, item No. 650970) with 200 μL of cell culture medium. The single cells generated MCSs ca. 400 μm in semidiameter at day 5 with 5% CO_2 in air at 37 °C. After formation of MCSs, the MCSs were treated with the compounds ([Ir] = 0-100 μM; DMSO content <2% v/v) for 2 h. Then the medium was replaced carefully by fresh medium. The MCSs were exposed blue light irradiation (20 min, 465 nm), then the MCSs were further incubated for 46 h. The cytotoxicities of the complexes toward MCSs were determined by ATP concentration measurements using the CellTiter-Glo® 3D Cell Viability Assay (Promega). After 60 min of incubation, MCSs were carefully transferred into black-sided, flat-bottomed 96-well plates and pipette-mixed for luminescence measurement on a Promega Glo-max Multi Microplate Reader.

8 spheroids were used for each condition studied. IC_{50} values were determined as duplicates of 8 spheroids in three independent sets of experiments and their standard deviations were calculated as above.

Detection of Intracellular $^1O_2$

Intracellular ROS generation by **Ir1** or **Ir1-HSA** after irradiation was detected using a red fluorescence cellular reactive oxygen species detection kit (ab186027, abcam). Firstly, the cultured cancer cells (10^5 cells) were incubated with 100 μL/well of the ROS probe working solution for 1 h in the incubator, then treated with **Ir1** or **Ir1-HSA** in the dark for 2 h, and washed with PBS. The ‘light’ plate was subjected to blue light irradiation for 5 min. The intensity of the red fluorescence was measured on a microplate reader (Promega) immediately. The excitation wavelength of the ROS probe was 520 nm, and the fluorescence was collected at 590-625 nm.

Table S1. Photophysical properties of **Ir1** and **Ir1-HSA** in air-saturated methanol-PBS solution (1:1, v/v). The standard used was $[Ru(bpy)_3]^{2+}$ (Φ_{em} = 0.028, $\Phi(^1O_2)$ = 0.22).

Compound	Lifetime τ([ns])	Emission quantum yield Φ_{em}	$^1O_2$ quantum yield $\Phi(^1O_2)$
**Ir1**	182.7	0.001	0.06
**Ir1-HSA**	871.8	0.036	0.83

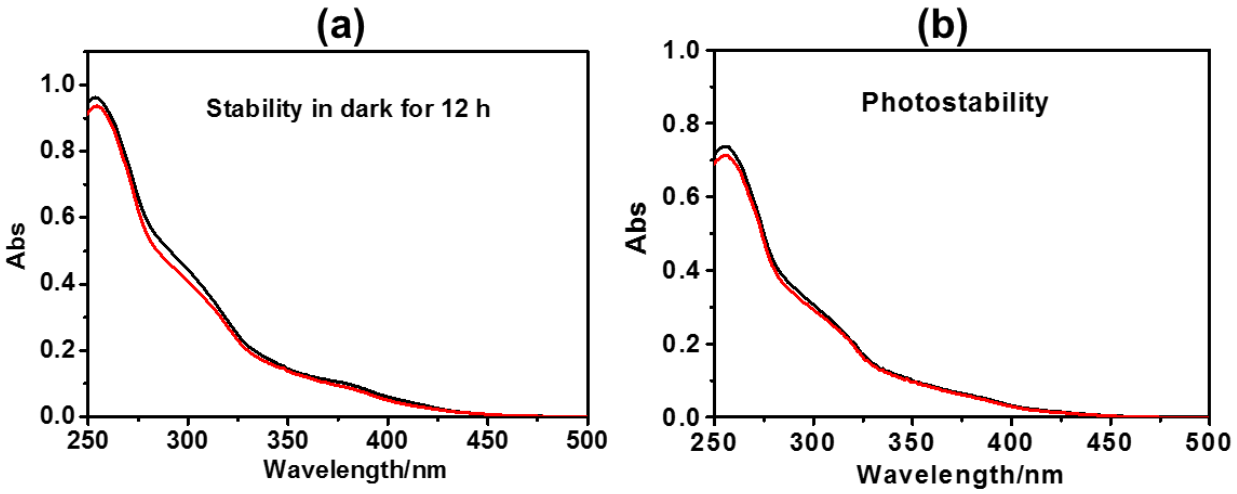

Figure S1. UV-vis absorption spectra of **Ir1** in PBS (containing 2% DMSO) (a) before and after 12 h in the dark, and (b) before and after blue light irradiation (465 nm) for 1 h.

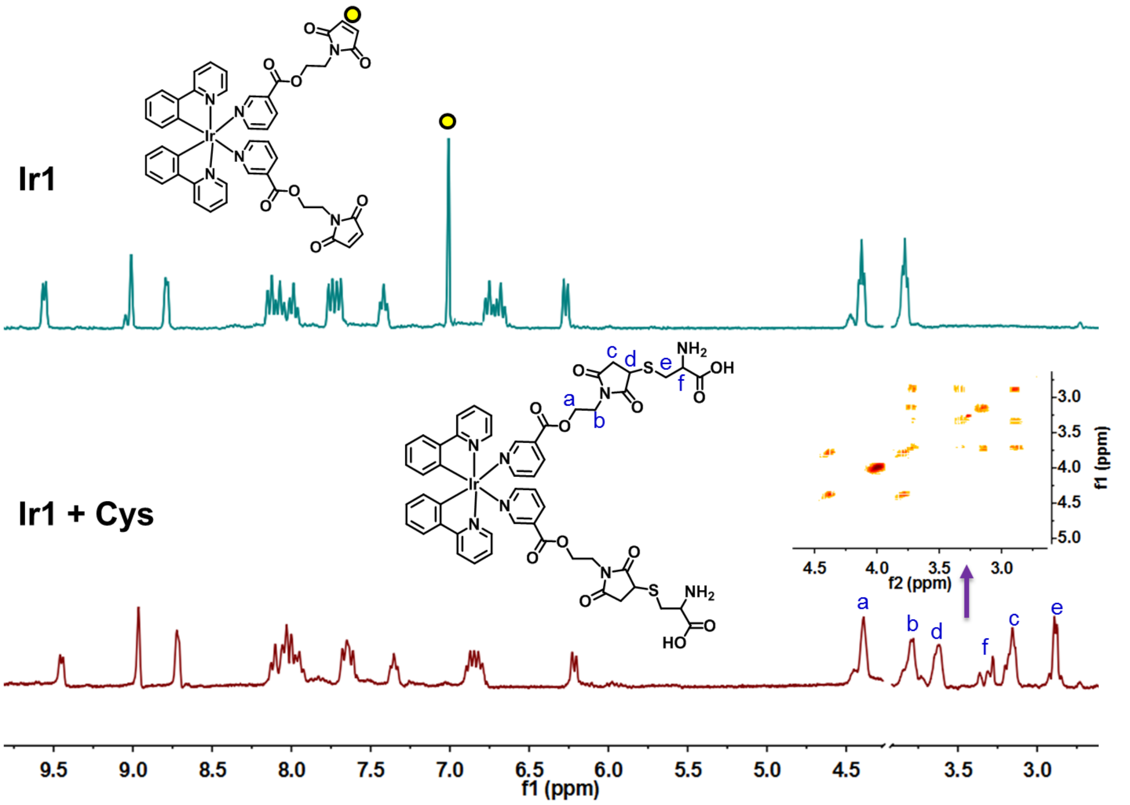

Figure S2. The change in the 400 Hz ^{1}H NMR spectrum of **Ir1** (2 mM) upon addition of 2 mol equiv of Cys in DMSO-d_6:D_2O 2:1 v/v after 30 min (Insect: 2D COSY spectrum of **Ir1** + Cys from 2.7 ppm to 5.0 ppm), showing the conjugation of both maleimide substituents to Cys sulfur.

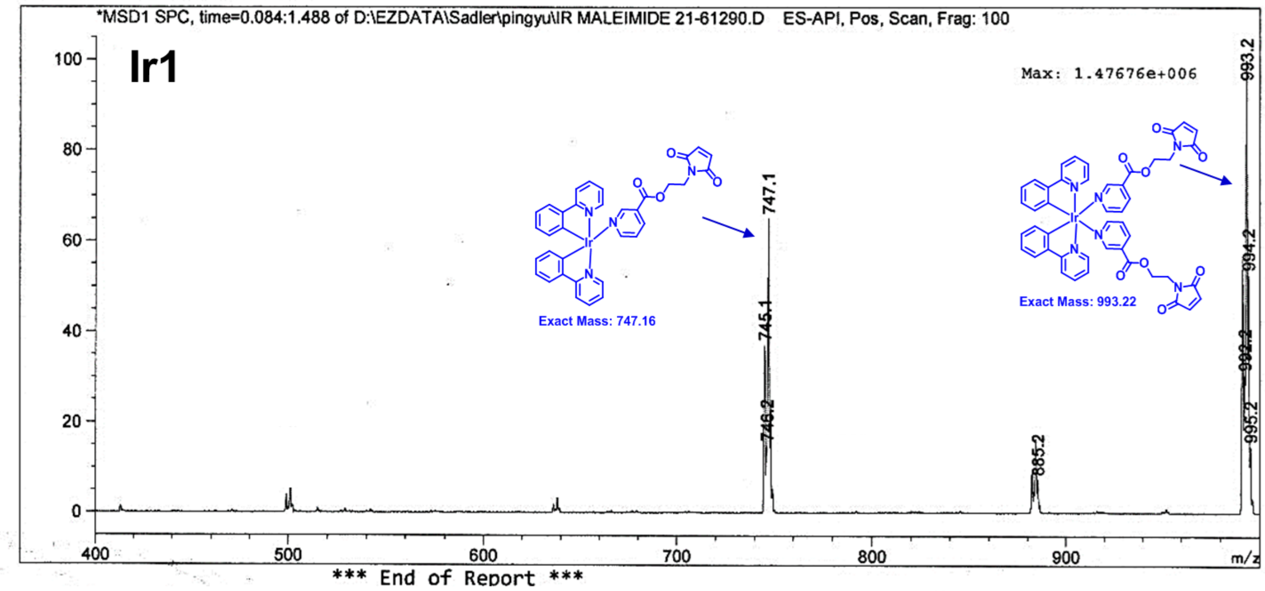

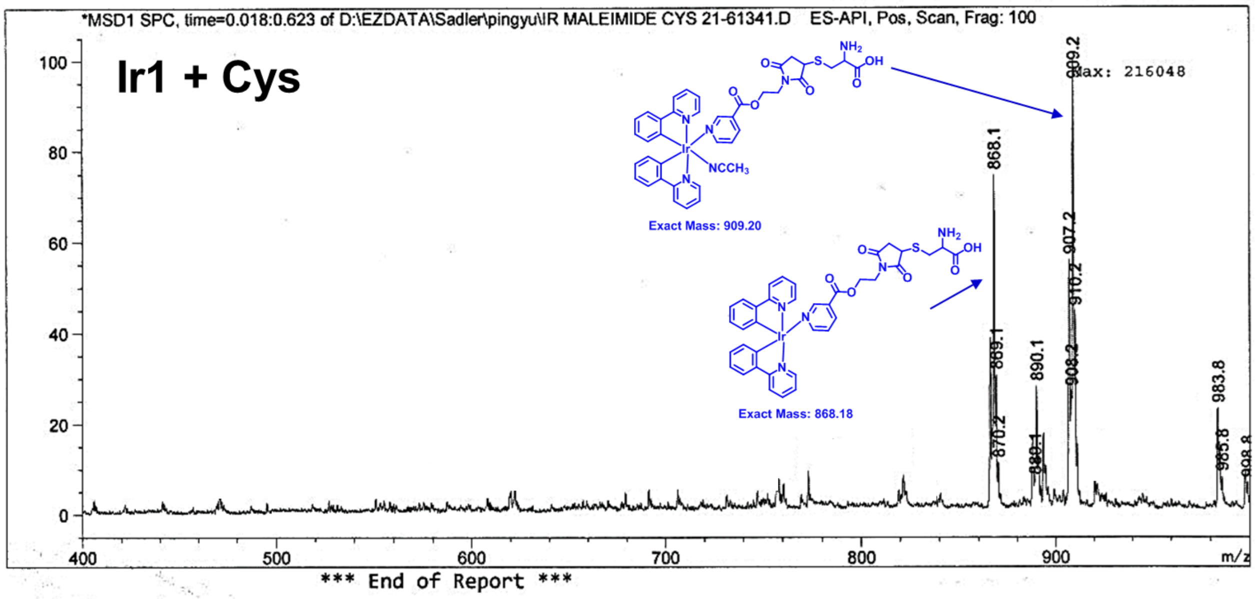

Figure S3. ESI-MS spectra of **Ir1** and the reaction mixture of **Ir1** with Cys in methanol.

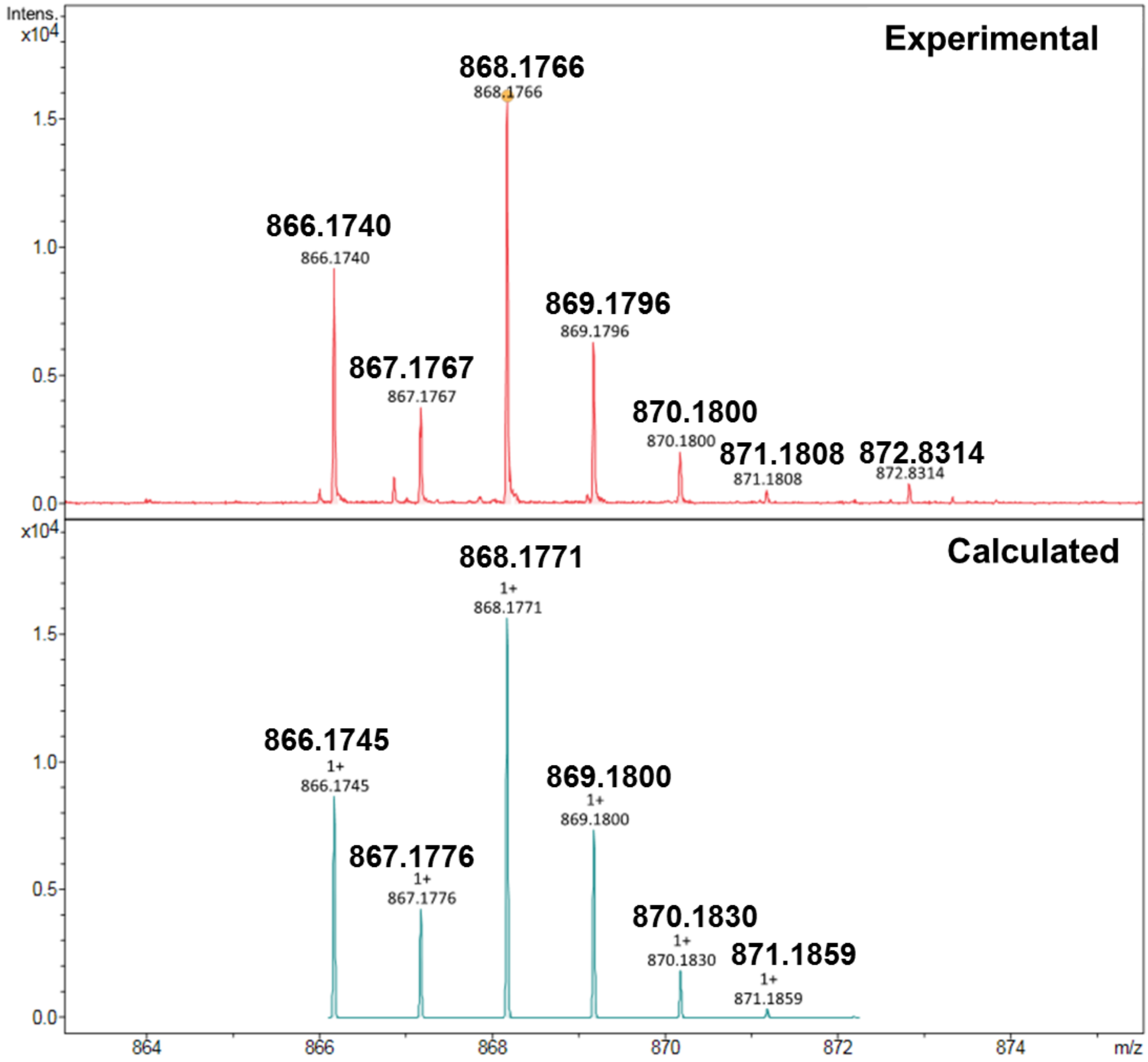

Figure S4. High-resolution mass spectrum (HR-MS) for the reaction mixture of **Ir1** and Cys; experimental and calculated spectra of peak with m/z = 868.1 ($C_{37}H_{33}IrN_5O_6S$) are shown.

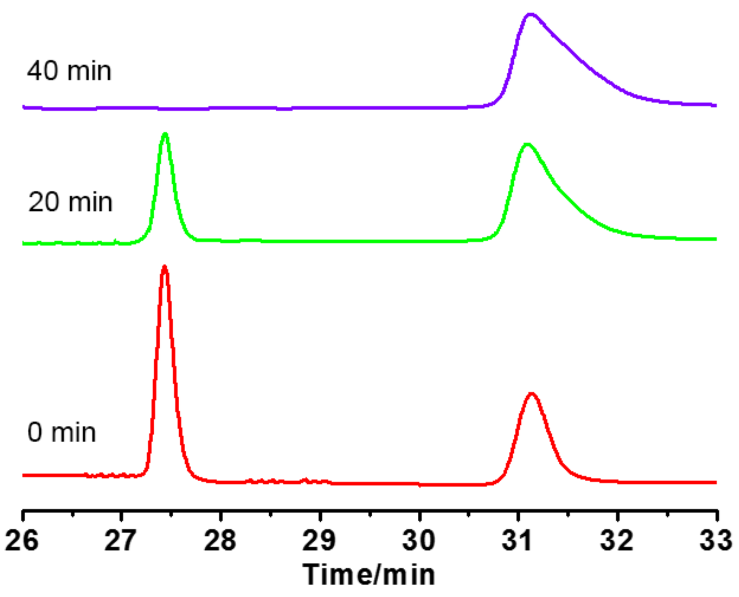

Figure S5. Time dependent HPLC traces for the reaction mixture containing **Ir1** (30 μM) and **HSA** (120 μM) in HPLC-grade water (UV detection at 280 nm).

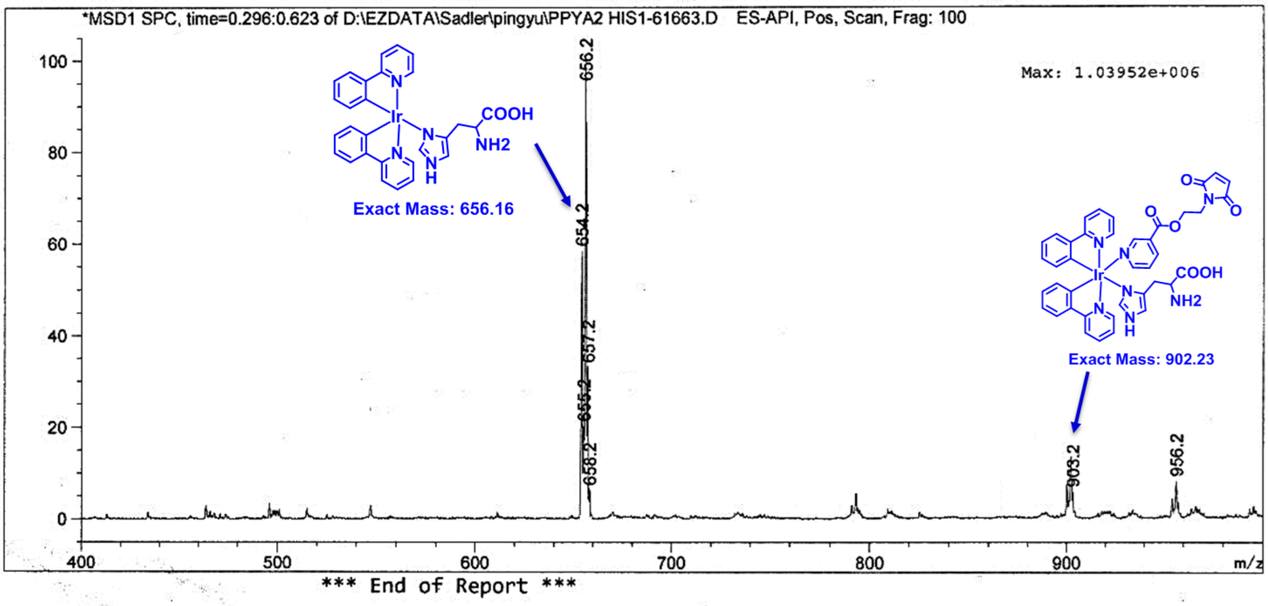

Figure S6. ESI-MS spectra of the reaction mixture of **Ir1** with His in methanol after 30 min.

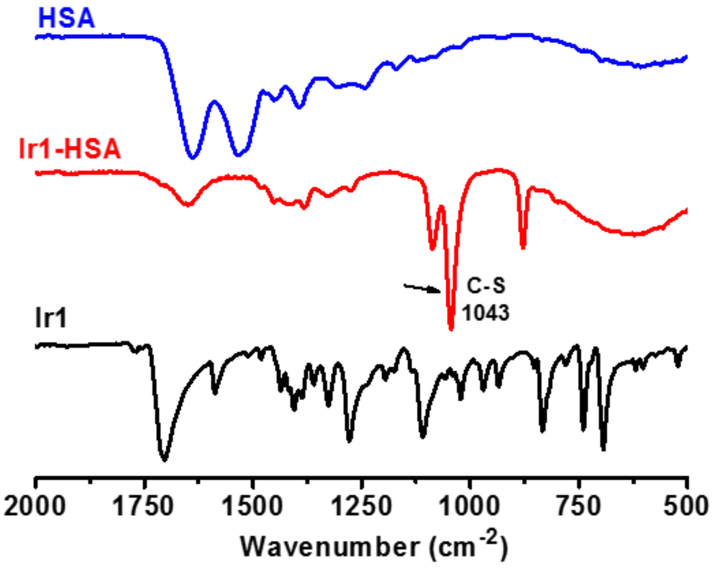

Figure S7. IR spectra of HSA, **Ir1** and **Ir1-HSA**.

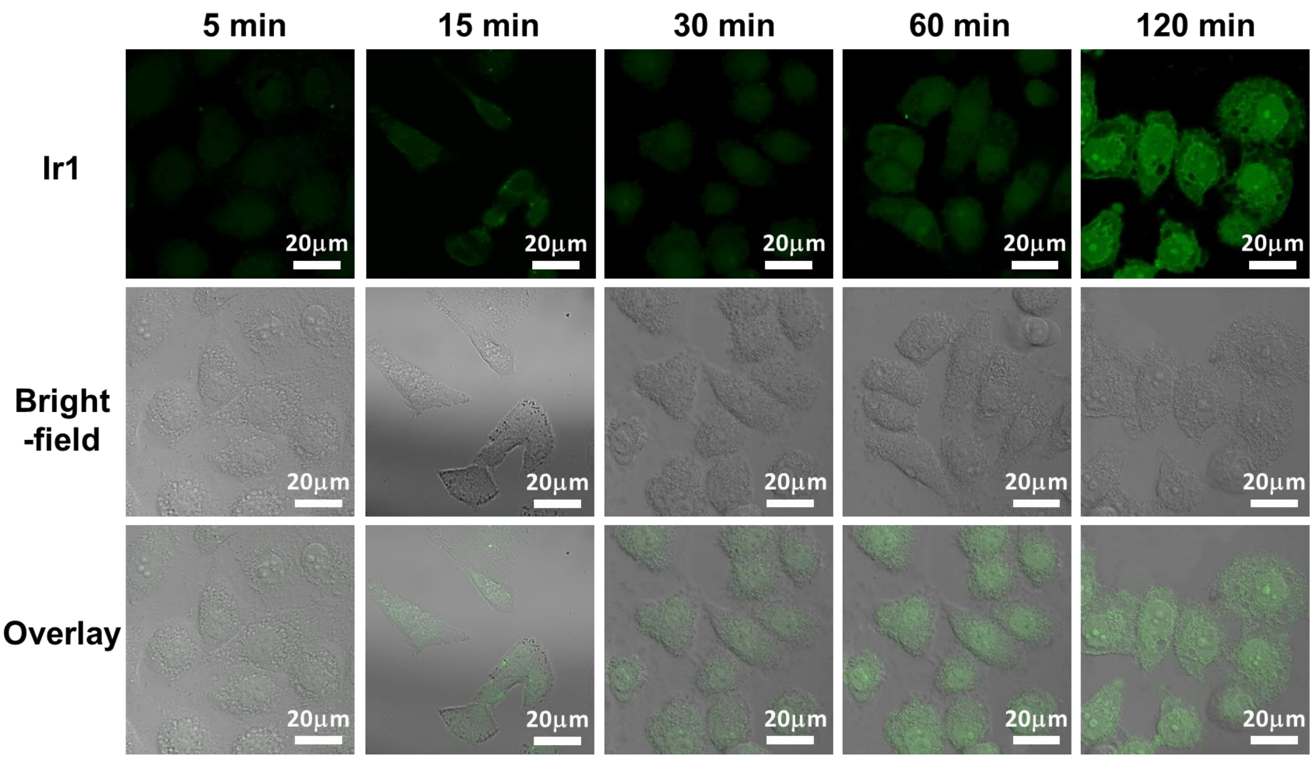

Figure S8. Confocal microscopy images of living A549 cells incubated with **Ir1** (5 μM) in real time (5 min, 15 min, 30 min, 60 min, 120 min); λ_{ex} = 405 nm, λ_{em} = 550 ± 20 nm.

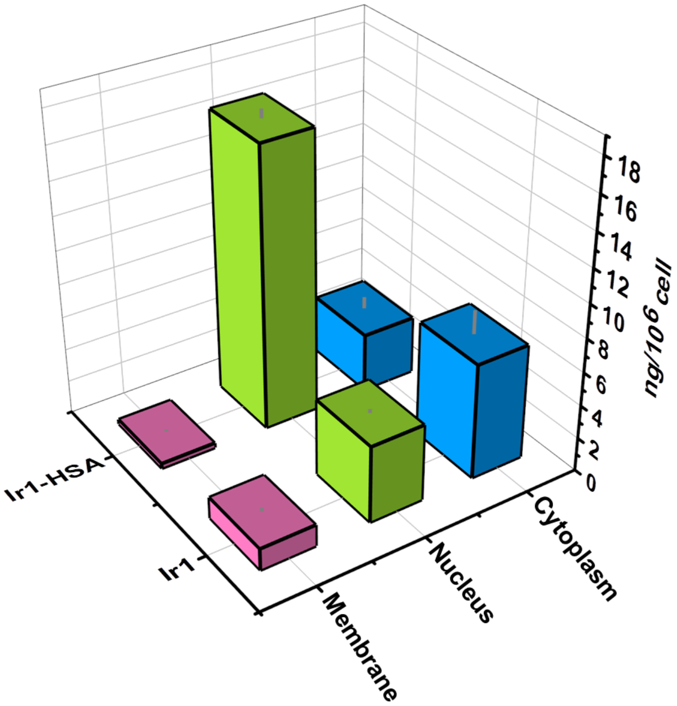

Figure S9. ICP-MS iridium concentrations in membrane, nucleus and cytoplasm of A549 human lung cancer cells after 2 h of exposure to **Ir1** and **Ir1-HSA** ([Ir] = 5 μM).

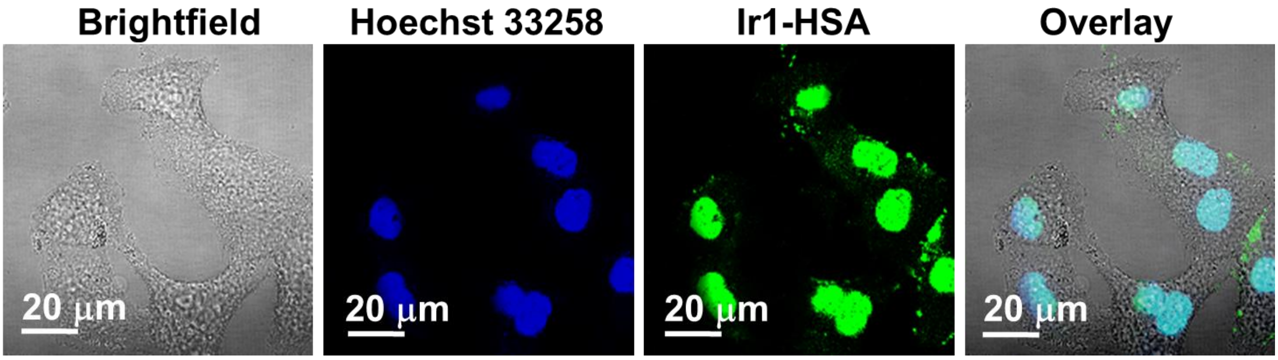

Figure S10. Confocal microscopy images of live A549 cells incubated with **Ir1-HSA** ([Ir] = 5 μM) for 2 h, and co-stained with Hoechst 33258 (500 nM) for 30 min. **Ir1-HSA**: λ_{ex} = 405 nm, λ_{em} = 550 ± 20 nm; Hoechst 33258: λ_{ex} = 405 nm, λ_{em} = 440 ± 20 nm; scale bar: 20 μm.

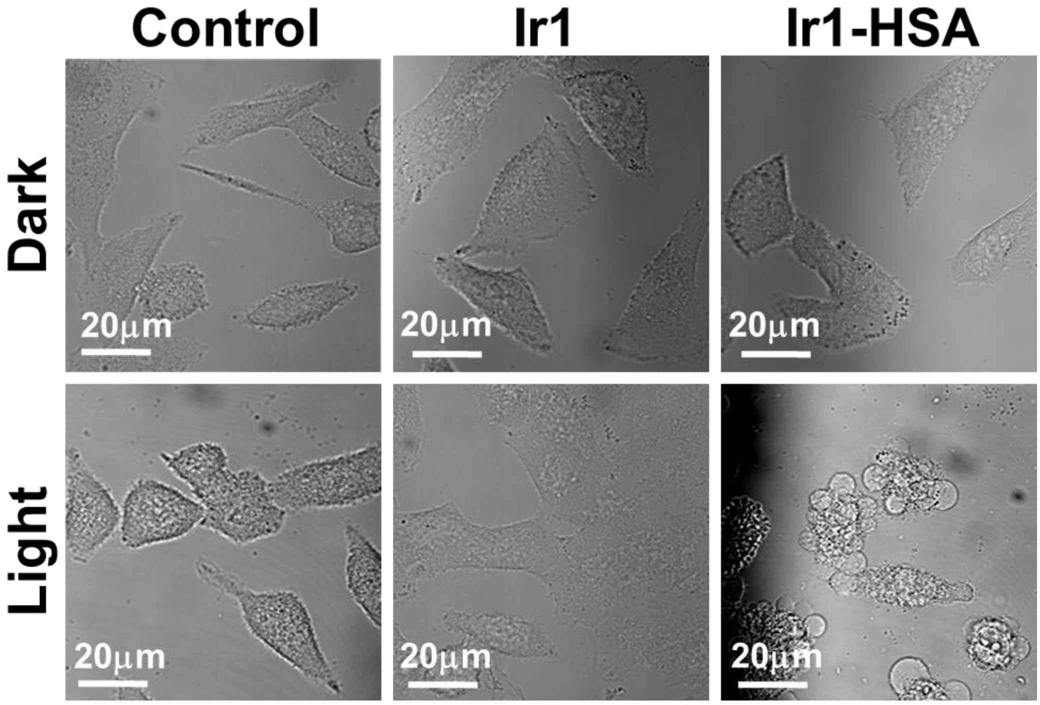

Figure S11. Brightfield images of control A549 cells, **Ir1** and **Ir1-HSA** treated cells ([Ir] = 5 μM, 2 h) in the dark and after irradiation (465 nm, 20 min).

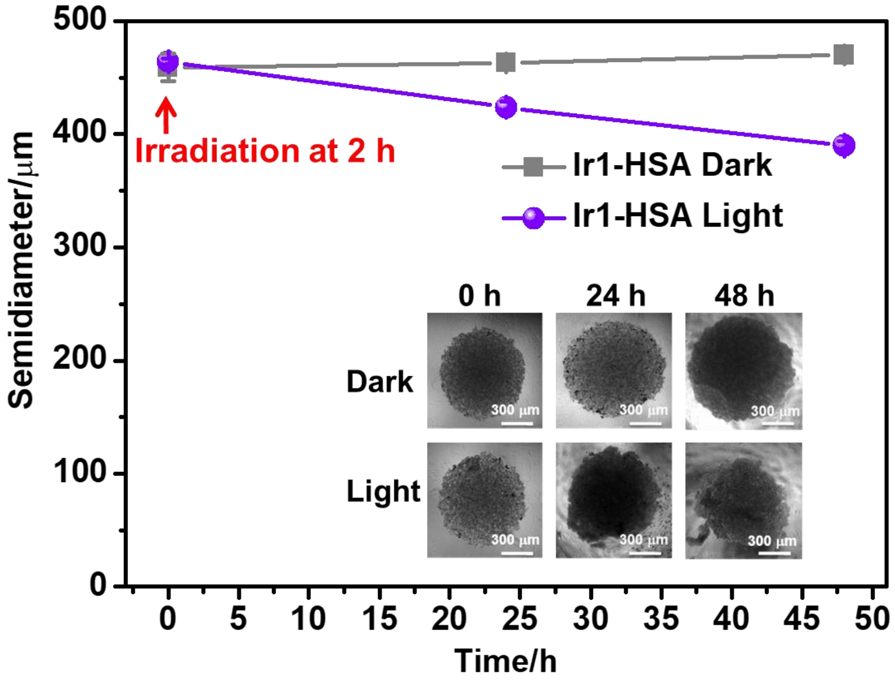

Figure S12. Growth curves for A549 3D MCSs treated for 2 h with **Ir1-HSA** ([Ir] = 5 μM) in the dark or followed by 20 min irradiation with 465 nm blue LEDs (5.76 J/cm^2); the inset pictures show brightfield images of the A549 3D MCSs at 0 h, 24 h and 48 h after light irradiation.

References

[1] G. Mantovani, F. Lecolley, L. Tao, D. M. Haddleton, J. Clerx, J. Cornelissen, K. Velonia, *J. Am. Chem. Soc.,* **2005**, *127*, 2966-2973.

[2] Y. Chen, L. Qiao, L. Ji, H. Chao, *Biomaterials,* **2014**, *35*, 2-13.

[3] a) A. J. Stewart, C. A. Blindauer, S. Berezenko, D. Sleep, D. Tooth, P. J. Sadler, *Febs Journal*, **2005**, *272*, 353-362; b) V. Pichler, J. Mayr, P. Heffeter, O. Dömötör, É. A. Enyedy, G. Hermann, D. Groza, G. Köllensperger, M. Galanksi, W. Berger, B. K. Keppler, *Chem. Commun.*, **2013**, *49*, 2249-2251.

[4] V. Vichai, K. Kirtikara, *Nature Protoc.* **2006**, *1*, 1112-1116.

[5] B. S. Howerton, D. K. Heldary, E. C. Glazer, *J. Am. Chem. Soc.,* **2012**, *134*, 8324-8327.
